# Academic demands and resources as predictors of emotional exhaustion and academic engagement in Chilean university students

**DOI:** 10.3389/fpsyg.2026.1885652

**Published:** 2026-06-25

**Authors:** Ricardo Jorquera-Gutiérrez, Felipe Guerra-Diaz, Letmy Martinez-Luna

**Affiliations:** Department of Psychology, Universidad de Atacama, Copiapó, Chile

**Keywords:** academic demands, academic engagement, academic resources, emotional exhaustion, study demands-resources, university student

## Abstract

This cross-sectional, non-experimental, correlational study tested a structural equation model based on Demands-Resources Theory (Demerouti et al.) applied to the university context, to examine the associations of academic demands (mental demands, work style, emotional demands, conflict with lecturers, and career choice anxiety) and academic resources (support from lecturers, possibility of personal development, information, feedback, and perceived control) with emotional exhaustion and academic engagement. A total of 424 university students from Chile participated. The model, estimated using WLSMV, showed adequate fit: χ^2^ = 2,604, *p* < 0.001; CFI = 0.964; TLI = 0.962; RMSEA = 0.050, 90% CI [0.048, 0.053]; SRMR = 0.069. Academic demands were positively associated with emotional exhaustion (*β* = 0.88, *p* < 0.001, R^2^ = 0.78). In contrast, academic resources were positively associated with engagement (*β* = 0.72, *p* < 0.001, R^2^ = 0.52). Additionally, a significant negative covariance was observed between resources and demands (*β* = −0.35, *p* < 0.001). The findings support the applicability of the SD-R model in academic contexts and suggest that strengthening institutional resources may help reduce student burnout risk and promote academic engagement.

## 1 Introduction

Over the past decades, the psychological well-being of university students has become an increasingly important topic in educational and health psychology because of the high prevalence of academic stress, exhaustion, and mental health problems documented in higher education worldwide (Auerbach et al., 2018). The transition to university involves a reorganization of cognitive, emotional, and social demands—such as high academic workloads, evaluative pressure, self-regulation requirements, and performance expectations—that may exceed the resources available to students (Salmela-Aro and Upadyaya, 2014). When this occurs, the risk of deterioration in psychological well-being increases, with consequences for academic performance, dropout intentions, and retention in higher education (Hahn et al., 2025; González et al., 2025). In this context, emotional exhaustion and academic engagement emerge as two central constructs for understanding student well-being.

Emotional exhaustion constitutes the core of academic burnout and is defined as a persistent feeling of emotional, mental, and physical fatigue stemming from the demands of studying (Schaufeli et al., 2002). In university populations, this dimension has proven particularly relevant, given that other classical dimensions of occupational burnout—depersonalization and reduced personal accomplishment—tend to manifest less consistently among students (Domínguez-Lara, 2013; Martínez and Marques Pinto, 2005). In Latin American contexts, emotional exhaustion has been positively associated with anxiety, depression, and test anxiety, and negatively associated with academic self-efficacy (Domínguez-Lara, 2013; Ramos et al., 2005).

In contrast, academic engagement represents a positive, persistent psychological state related to studying, characterized by vigor, dedication, and absorption (Schaufeli et al., 2002). Evidence indicates that engagement is associated with better academic outcomes, lower dropout intention, and greater satisfaction with studies (Bakker et al., 2015; Tuominen-Soini and Salmela-Aro, 2014).

Within this framework, the Job Demands-Resources Model (JD-R), proposed by Demerouti et al. (2001), explains well-being and ill-being in organizational contexts. The model distinguishes between demands—aspects of the environment that require sustained effort and generate psychological costs—and resources—aspects that facilitate the achievement of goals, reduce the impact of demands, and promote personal growth (Bakker and Demerouti, 2007). Based on this distinction, the JD-R posits two complementary processes: a health impairment process, whereby high demands lead to exhaustion, and a motivational process, in which resources foster engagement and performance (Bakker and Demerouti, 2017).

Building on the JD-R, this framework has been adapted to the educational context, giving rise to Study Demands-Resources (SD-R) models. These models hold that the university experience can be organized analogously to work, given that studying involves structured, goal-oriented activities that are institutionally evaluated (Salanova et al., 2010). Bakker and Mostert (2024) integrated accumulated evidence into an SD-R Theory that conceptualizes academic demands—workload, time pressure, cognitive and emotional demands—and study resources—instructor support, feedback, autonomy, development opportunities—as direct and combined predictors of emotional exhaustion and engagement, maintaining the two core processes of the JD-R and additionally proposing buffering and boosting effects of resources on demands.

Prior research has shown that academic demands are positively associated with emotional exhaustion and other indicators of ill-being, while study resources are negatively related to burnout and positively related to engagement (Gusy et al., 2016; Lesener et al., 2020; Salmela-Aro et al., 2022). Additionally, emotional exhaustion and engagement have been found to act as mediators between academic environment characteristics and student outcomes such as life satisfaction, academic performance, and dropout intention (Salanova et al., 2010; Lesener et al., 2020).

However, despite the theoretical development of the SD-R framework, empirical studies examining its dual process remain scarce and have been conducted primarily in European and North American contexts, with limited Latin American representation (Bakker and Mostert, 2024). Lesener et al. (2020), in a sample of 5,660 German university students, confirmed that demands predict burnout (*β* = 0.50), while resources predict engagement (*β* = 0.70) and reduce burnout (*β* = −0.35), yet noted the need to test the model in other cultural and educational contexts. In the region, studies on burnout and engagement among university students have predominantly adopted descriptive or correlational designs, analyzing these variables in isolation or as pairs, without testing the dual-process model through structural equation modeling (Hederich-Martínez and Caballero-Domínguez, 2016; Mamani-Benito et al., 2024). This gap limits the evaluation of the joint predictive capacity of the model and the specific magnitude of each pathway in Latin American university populations.

In the Chilean context, research on well-being among university students has been predominantly oriented toward studying the prevalence of individual mental health symptoms. A scoping review with meta-analysis including 32 studies published in Chile reported prevalences of psychological distress between 22.9 and 40.7%, depressive symptoms between 16.5 and 38.8%, and anxiety symptoms between 16.5 and 23.7%, without identifying studies that evaluated the effectiveness of interventions aimed at promoting well-being in this population (Martínez et al., 2021). National studies have further documented that more than 45% of Chilean university students present risk symptoms associated with depression, anxiety, or stress (Barrera-Herrera and San Martín, 2021). Nevertheless, available evidence on the application of the SD-R framework in the Chilean university context is scarce, limiting the possibility of systematically identifying which characteristics of the university environment operate as risk factors and which as protective factors for student well-being.

The present study makes a twofold contribution. Theoretically, it constitutes one of the first studies in the Chilean university context to empirically verify the dual process of the SD-R framework through a model that integrates academic demands and resources as simultaneous predictors of emotional exhaustion and academic engagement. In applied terms, its findings can guide the design of institutional interventions aimed both at reducing risk factors associated with well-being deterioration and at strengthening resources that promote engagement, with implications for student retention and dropout prevention in Chilean higher education.

Accordingly, this study aimed to evaluate the fit and predictive pathways of a structural equation model designed to explain emotional exhaustion and academic engagement in Chilean university students based on the Study Demands-Resources Model. Based on the SD-R framework, two hypotheses were formulated: H1, academic demands would be positively associated with emotional exhaustion; and H2, academic resources would be positively associated with academic engagement. In addition, given previous theoretical and empirical developments, we expected demands and resources to be negatively related at the latent level.

## 2 Materials and methods

### 2.1 Research design

A non-experimental, cross-sectional, correlational design was employed, as the study variables were measured at a single point in time.

### 2.2 Participants

Purposive non-probability sampling was used. The sample comprised 424 Chilean university students from various degree programs at a university in northern Chile. To be included in the study, participants had to be actively and officially enrolled in an undergraduate degree program at the host institution; individuals who were not matriculated or were only auditing courses were excluded. Their mean age was 19.8 years (SD = 2.16). By gender, 65.6% (*n* = 278) were women, 33.3% (*n* = 141) were men, and 1.2% (*n* = 5) identified with another gender. By field of study, 39.6% (*n* = 168) were enrolled in humanities, education, or social sciences programs; 37.7% (*n* = 160) in health sciences or medicine programs; 22.2% (*n* = 94) in engineering programs; and 0.5% (*n* = 2) did not report their field. All participants were undergraduate students. Regarding year of study, 57.3% (*n* = 243) were in their first year, 28.1% (*n* = 119) were in their second year, and 14.6% (*n* = 62) were in their third year or above.

### 2.3 Instruments

Three instruments were used for measurement.

First, a Spanish translation of the University Demand-Resource Questionnaire (UDRQ; Jagodics and Szabó, 2023) was administered. This instrument assesses two broad latent factors through 34 items in a 6-point Likert format. The Academic Demands factor (17 items) covers five specific dimensions: mental demands, work style, emotional demands, conflict with lecturers, and career choice anxiety. The Academic Resources factor (17 items) assesses support from lecturers, possibility of personal development, information, feedback, and perceived control. Evidence of adequate psychometric properties has been reported for this instrument. The resource subscales showed Cronbach’s alpha coefficients ranging from 0.74 to 0.90 and average variance extracted (AVE) values ranging from 0.49 to 0.69. For the demand subscales, Cronbach’s alpha coefficients for the five subdimensions ranged from 0.75 to 0.87, and AVE values ranged from 0.45 to 0.69 (Sun et al., 2024).

Second, the Emotional Exhaustion Scale (EES), developed by Ramos et al. (2005) and psychometrically adapted by Domínguez-Lara (2013), was administered. This scale consists of 10 unidimensional items assessing perceived exhaustion over the previous 12 months of student life, using a 5-point Likert response scale. Previous research has supported its unidimensional structure and reported a reliability coefficient of 0.87 (Domínguez-Lara, 2014).

Finally, academic engagement was measured using the student version of the Utrecht Work Engagement Scale (UWES-S), specifically the 9-item short version (UWES-9S). This instrument assesses three interrelated dimensions: vigor (energy and mental resilience), dedication (enthusiasm and sense of meaning), and absorption (deep concentration). Prior research in Chile has confirmed that the 9-item version provides better fit indices (χ^2^/df = 2.316; CFI = 0.977; TLI = 0.952; RMSEA = 0.071) and reliability (McDonald’s *ω* between 0.67 and 0.83) compared to the 17-item version, while also demonstrating factorial invariance across gender and level of study (Jorquera Gutiérrez and Guerra Díaz, 2021).

### 2.4 Procedures

The data collection period was between September and November 2024. Participants were approached in their classrooms and invited to participate anonymously, voluntarily, and without compensation. Before instrument administration, the general aims of the study, its academic nature, and the exclusive use of the data for scientific purposes were explained. All participants provided informed consent, which explicitly stated the voluntary nature of participation, the right to withdraw at any time without consequences, data confidentiality, the absence of foreseeable risks, and the contact details of the research team.

The instruments were administered via an electronic form (Google Forms), accessed through a QR code. Informed consent was presented in the first section of the form, and its acceptance was a mandatory requirement to proceed with the survey. No sensitive information or data that would allow direct or indirect identification of participants was collected, and responses were stored and analyzed in aggregate form.

Given that the study was classified as minimal risk, as it was based on self-administered instruments that did not collect sensitive information or personal identification data, formal approval from an institutional ethics committee was not required, in accordance with international standards for educational research and exemption policies for non-invasive studies in adult populations. This is consistent with Chilean legislation on scientific research involving human beings and with the principles of the Declaration of Helsinki.

### 2.5 Statistical analysis procedure

Descriptive analyses (measures of central tendency and dispersion) and correlational analyses of the study variables were conducted. Reliability of the scales was estimated using Cronbach’s alpha coefficient and McDonald’s omega. Missing data were first inspected before model estimation, because the proportion of missing responses was low and no systematic pattern suggesting substantial bias was detected. All descriptive analyses were performed in SPSS 25.

Structural equation modeling analyses were conducted in R (RStudio interface) using the lavaan package. Due to the ordinal nature of the data and the lack of multivariate normality, the robust weighted least squares estimator with mean and variance adjustment (WLSMV) was employed.

Overall model fit was evaluated using the chi-square statistics, the comparative fit index (CFI), the Tucker-Lewis index (TLI), the root mean square error of approximation (RMSEA), together with its 90% confidence interval, and the standardized root mean square residual (SRMR). Values above 0.95 for CFI and TLI, and values below 0.06 for RMSEA, were considered indicators of good fit (Hu and Bentler, 1999), while SRMR values below 0.08 were interpreted as acceptable.

## 3 Results

### 3.1 Descriptive statistics and correlations

Descriptive results showed that emotional exhaustion had a mean of 3.52 (SD = 0.89), while academic engagement had a mean of 3.79 (SD = 1.13). Within the latter construct, the dimension with the highest score was absorption (M = 3.72; SD = 1.23). Among academic resources, the dimension with the highest mean score was the possibility of personal development (M = 4.91; SD = 0.92), while among academic demands, the highest was mental demands (M = 4.25; SD = 1.19).

The relationships between the variables showed that emotional exhaustion correlates negatively with academic engagement (*r* = −0.24; *p* < 0.01), both overall and with each of its dimensions. It also correlates negatively with all academic resources measured, with coefficients between −0.19 and −0.31 (*p* < 0.01). Additionally, emotional exhaustion correlates positively with academic demands, with coefficients between 0.30 and 0.65 (*p* < 0.01).

In terms of reliability, the assessment instruments used showed adequate Cronbach’s alpha coefficients, ranging between 0.71 and 0.91 (Table 1).

**Table 1:** Descriptive statistics, alpha, and correlations of study variables.

Variables	M	SD	*α*	ꙍ	AVE	1	2	3	4	5	6	7	8	9	10	11	12	13	14
1. Emotional Exhaustion	3.52	0.89	0.91	0.91	0.52														
2. Engagement	3.79	1.13	0.91	0.91		−0.24**													
3. Vigor	3.01	1.36	0.80	0.80	0.58	−0.27**	0.89**												
4. Dedication	4.63	1.25	0.86	0.85	0.66	−0.18**	0.86**	0.61**											
5. Absorption	3.72	1.23	0.78	0.78	0.54	−0.18**	0.91**	0.73**	0.68**										
6. Support from Lecturers	4.48	1.02	0.75	0.76	0.51	−0.31**	0.46**	0.38**	0.44**	0.42**									
7. Possibility of Development	4.91	0.92	0.85	0.85	0.59	−0.21**	0.60**	0.43**	0.66**	0.53**	0.63**								
8. Information	4.40	1.00	0.83	0.83	0.55	−0.19**	0.45**	0.36**	0.43**	0.40**	0.70**	0.70**							
9. Feedback	4.10	1.09	0.77	0.76	0.52	−0.20**	0.48**	0.43**	0.39**	0.45**	0.58**	0.56**	0.76**						
10. Perceived Control	4.31	1.13	0.82	0.82	0.60	−0.28**	0.47**	0.39**	0.43**	0.42**	0.49**	0.53**	0.48**	0.48**					
11. Mental Demands	4.25	1.19	0.81	0.81	0.60	0.65**	−0.14**	−0.19**	−0.06	−0.11*	−0.16**	−0.02	−0.05	−0.14**	−0.17**				
12. Work Style	3.88	1.13	0.80	0.80	0.50	0.61**	−0.09	−0.14**	−0.03	−0.07	−0.15**	−0.07	−0.11*	−0.07	−0.11*	0.60**			
13. Emotional Demands	3.93	1.34	0.71	0.70	0.44	0.59**	−0.23**	−0.22**	−0.21**	−0.18**	−0.24**	−0.18**	−0.11*	−0.14**	−0.26**	0.66**	0.45**		
14. Conflict with Lecturers	2.45	1.30	0.75	0.75	0.52	0.30**	−0.21**	−0.20**	−0.21**	−0.14**	−0.36**	−0.29**	−0.29**	−0.15**	−0.20**	0.26**	0.32**	0.27**	
15. Career Choice Anxiety	3.64	1.40	0.79	0.80	0.57	0.35**	−0.27**	−0.23**	−0.22**	−0.26**	−0.16**	−0.18**	−0.08	−0.08	−0.20**	0.40**	0.27**	0.53**	0.17**

**Correlation is significant at the 0.01 level (two-tailed). *Correlation is significant at the 0.05 level (two-tailed).

#### 3.1.1 Measurement model

To evaluate the assumption of multivariate normality, the Mardia coefficient test was applied. The results indicated a multivariate skewness (Coefficient = 531, z = 3,753, X2 = 924,804, df = 4, *p* < 0.001) and significant multivariate kurtosis (Coefficient = 3,149, z = 46.9, *p* < 0.001). Due to this failure to assume normality, the use of robust estimation methods for subsequent analyses is warranted.

The evaluation of the measurement model was conducted in two stages: first, a first-order analysis of the individual dimensions, and subsequently, a second-order analysis of the core constructs (Resources, Demands, and Engagement). As shown in Table 2, all observed variables loaded significantly on their respective first-order factors (*p* < 0.001). In most cases, standardized loadings exceeded 0.60, although somewhat lower values were observed for specific indicators (e.g., R12 for feedback, D11 for emotional demands, and CE2 for emotional exhaustion). Taken together, these results support the internal structure of the instruments and provide evidence of convergent validity at the indicator level. In addition, the reliability and validity indices reported in Table 1 show generally adequate internal consistency across scales and subscales, with alpha and omega coefficients in acceptable-to-high ranges and average variance extracted values that were mostly around or above the conventional 0.50 criterion, although emotional demands showed a comparatively lower AVE. This pattern suggests overall satisfactory convergent validity, while also indicating that some subdimensions should be interpreted with greater caution. At the construct level, the correlation pattern among subscales was theoretically coherent and did not suggest problematic overlap among the higher-order dimensions, supporting an acceptable level of discriminant validity for the proposed model.

**Table 2:** First-Order measurement model: standardized factor loadings.

Dimension / Item	λ (Stand.)	SE	z value	*p*
Resources (Dimensions)				
Lecturers: R1 / R2 / R3	0.67/0.77/0.69	0.07	13.64	<0.001
Development: R4 / R5 / R6 / R7	0.78/0.81/0.78/0.71	0.06	17.17	<0.001
Information: R8 / R9 / R10 / R11	0.88/0.66/0.67/0.75	0.07	13.47	<0.001
Feedback: R12 / R13 / R14	0.59/0.72/0.86	0.10	11.17	<0.001
Control: R15 / R16 / R17	0.77/0.79/0.75	0.07	14.77	<0.001
Demands (Dimensions)				
Mental demands: D1 / D2 / D3	0.65/0.85/0.79	0.12	11.09	<0.001
Work Style: D4 / D5 / D6 / D7	0.64/0.81/0.76/0.62	0.11	11.39	<0.001
Emotional demands: D8 / D10 / D11	0.77/0.66/0.57	0.08	11.62	<0.001
Conflict with lecturers: D12 / D13 / D14	0.60/0.75/0.76	0.16	8.74	<0.001
Career choice anxiety: D15 / D16 / D17	0.71/0.79/0.75	0.14	8.56	<0.001
Emotional exhaustion				
CE1 to CE10 (Range)	0.55–0.87	0.07	11.18	<0.001
Engagement (Dimensions)				
Vigor: EN1 / EN2 / EN5	0.66/0.81/0.79	0.10	12.55	<0.001
Dedication: EN3 / EN4 / EN7	0.80/0.87/0.78	0.07	14.60	<0.001
Absorption: EN6 / EN8 / EN9	0.80/0.73/0.67	0.06	12.81	<0.001

The goodness of fit of the measurement models corresponding to the four dimensions analyzed was independently evaluated. The results showed an adequate fit for the second-order engagement models (x^2^ = 23; *p* = 0.522; CFI = 1,000; RMSEA = 0.000, 95% CI [0.000, 0.037]) and resource factors (x^2^ = 98.4; *p* = 0.851; CFI = 1,000; RMSEA = 0.000, 95% CI [0.000, 0.014]). On the other hand, the demand factors dimension showed a satisfactory adjustment (x^2^ = 260; *p* < 0.001; CFI = 0.967; RMSEA = 0.062, 95% CI [0.053, 0.071]), while the emotional exhaustion dimension also exhibited optimal fit indices (x^2^ = 28.8; *p* = 0.760; CFI = 1,000; RMSEA = 0.062, 95% CI [0.000, 0.025]).

Regarding the second-order structure (Table 3), the results confirm that the higher-order constructs are adequately defined by their subdimensions. Engagement showed high factor loadings for vigor (*β* = 0.89), dedication (*β* = 0.91), and absorption (*β* = 0.95). Similarly, resources and demands demonstrated a consistent structure, with all dimensions contributing significantly to their respective latent factors.

**Table 3:** Second-order measurement model.

Latent construct	Indicator dimension	β (Stand.)	SE	z value	*p*
Resources	Lecturers	0.92	—	—	—
	Development	0.88	0.08	10.79	<0.001
	Information	0.85	0.08	13.34	<0.001
	Feedback	0.86	0.09	9.80	<0.001
	Perceived control	0.76	0.10	9.57	<0.001
Demands	Mental demands	0.83	—	—	—
	Work Style	0.75	0.14	6.90	<0.001
	Emotional demands	0.91	0.14	9.99	<0.001
	Career choice anxiety	0.56	0.13	6.73	<0.001
	Conflict with lecturers	0.51	0.09	6.04	<0.001
Engagement	Vigor	0.89	—	—	—
	Dedication	0.91	0.12	9.29	<0.001
	Absorption	0.95	0.12	10.94	<0.001

#### 3.1.2 Structural model

The results indicate that the proposed structural model showed adequate overall fit to the data (χ^2^ = 2,604, *p* < 0.001; χ^2^/df = 2.07; CFI = 0.964; TLI = 0.962; RMSEA = 0.050, 95% CI [0.048, 0.053]; SRMR = 0.069).

The standardized path linking academic demands with emotional exhaustion was positive (*β* = 0.88, *p* < 0.01), indicating that higher perceived academic demands tended to co-occur with greater emotional exhaustion. This latent association accounted for 78% of the variance in emotional exhaustion (R^2^ = 0.78).

**Figure 1:**
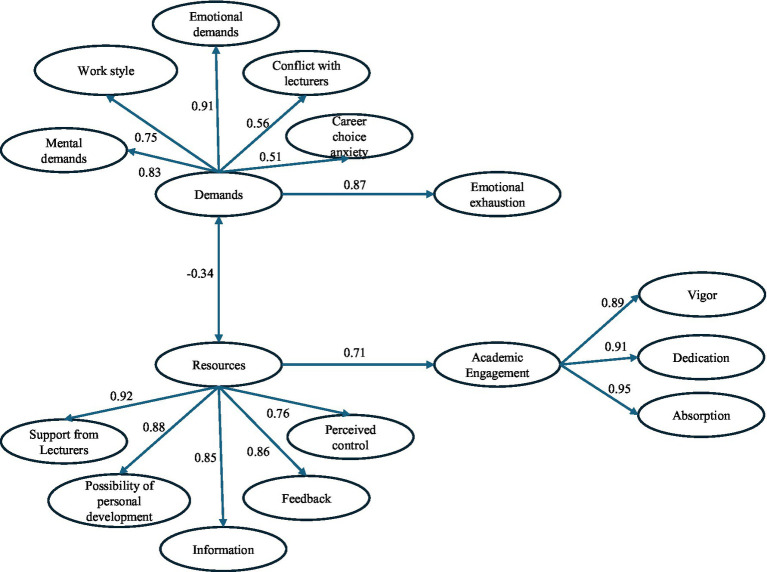
Structural model SD-R, emotional exhaustion and academic engagement.

In turn, academic resources were positively associated with academic engagement (*β* = 0.72, *p* < 0.01), explaining 51.7% of its variance. Additionally, a significant negative covariance between resources and demands was identified (*β* = −0.35, *p* < 0.01), suggesting that students who perceived more supportive and resourceful academic environments also tended to report lower levels of demand. Although the global latent effects were strong, inspection of the first- and second-order loadings suggests that not all indicators contributed equally to their higher-order constructs. Mental demands and emotional demands appeared to be more salient within the demands domain than conflict with lecturers or career choice anxiety, whereas support from lecturers, development opportunities, information, feedback, and perceived control all made substantial contributions within the resources’ domain, with lecturer support standing out as especially relevant (Figure 1).

## 4 Discussion

The findings of the present study provide empirical support for the applicability of Study Demands-Resources Theory (SD-R) among Chilean university students. More specifically, the pattern of associations observed in the structural model was consistent with the health-impairment process linked to study demands and the motivational process linked to study resources (Demerouti et al., 2001; Bakker and Demerouti, 2007; Bakker and Mostert, 2024). Importantly, because the design was cross-sectional and non-experimental, these results should be interpreted as associations rather than causal effects. Even so, the magnitude of the association between academic demands and emotional exhaustion was notably high, which is consistent with the idea that sustained exposure to cognitively, emotionally, and organizationally demanding study conditions is closely intertwined with students’ feelings of depletion and overload (Bakker and Demerouti, 2017; Lesener et al., 2020).

The descriptive results help contextualize this pattern. Emotional exhaustion showed a mean above the midpoint of the response scale, suggesting a moderate level of fatigue in this sample rather than an isolated or marginal problem. At the same time, academic engagement also showed relatively favorable mean levels, with absorption representing the highest average among its dimensions, whereas vigor was comparatively lower. This combination suggests that many students remain psychologically invested in their studies but do so under conditions that may also be emotionally draining. In practical terms, students appear focused and interested yet not necessarily energized to the same degree. This coexistence of engagement and exhaustion is theoretically plausible within the SD-R framework, because positive involvement in study tasks does not automatically eliminate strain when demands remain high.

SD-R theory proposes that study demands may include both challenge-like characteristics that can stimulate learning and hindrance-like characteristics that obstruct progress (Bakker and Mostert, 2024). This distinction is useful for interpreting the unequal contribution of the demand indicators observed in the present study. Mental demands and emotional demands appeared to be especially prominent, suggesting that the burden experienced by students may be rooted less in overt conflict and more in the continuous cognitive effort and emotional regulation required to meet academic expectations. By contrast, conflict with lecturers and career choice anxiety, although relevant, contributed less strongly to the higher-order demands construct. This pattern may indicate that day-to-day academic strain is primarily shaped by the immediate workload and emotional pressure of studying, whereas relational conflict and vocational uncertainty operate as additional but less central stressors.

In the Chilean context, career choice anxiety may carry weight due to the economic pressure and expectations associated with educational investment, which increases the perceived risk of vocational error and fosters persistent worry (Bakker and Mostert, 2024; Valenzuela et al., 2021). This type of vocational uncertainty can affect the sense of purpose underlying academic effort, facilitating the emotional and cognitive distancing that characterizes burnout (Maslach et al., 2001). Similarly, conflicts with lecturers, linked to perceptions of injustice or low evaluative clarity, can increase emotional tension and hinder self-regulation of learning, intensifying exhaustion (Bakker and Mostert, 2024).

Regarding the motivational process, the positive association between academic resources and engagement is consistent with the view that resources are functional for goal attainment, facilitate learning, and promote sustained psychological investment in academic tasks (Bakker and Demerouti, 2007; Lesener et al., 2020). In the present study, the highest mean among resources corresponded to the possibility of personal development, suggesting that students generally perceived university as a setting with growth opportunities. Nevertheless, support from lecturers, information, feedback, and perceived control also made substantial contributions to the latent resource factor. This is important because it suggests that engagement is not linked only to broad developmental opportunities, but also to proximal instructional conditions that make academic work understandable, manageable, and meaningful in everyday practice.

The negative association between demands and resources suggests that academic environments perceived as more supportive also tend to be experienced as less taxing. This should not be interpreted as evidence that resources eliminate demands, but rather that they may shape how students appraise and manage them. For example, clear information, timely feedback, and a sense of control may reduce ambiguity and unnecessary strain, making demanding academic activities feel more manageable. From an applied perspective, this point is highly relevant because it implies that not all intervention efforts need to focus on reducing workload directly; strengthening the quality of the academic environment may also help attenuate the harmful side of demands.

International evidence suggests that the intensity of these relationships may vary according to individual characteristics and contextual conditions. For example, during the pandemic, gender differences were observed, with women reporting higher demands and exhaustion, and lower engagement, in university samples (Ghislieri et al., 2023). At the level of personal resources, JDR-based studies have shown that self-compassion can act as a protective resource by mediating the relationship between demands and burnout, and by promoting engagement (Lee et al., 2022). In addition, academic self-efficacy has been described as protective, but its effect may be weakened under conditions of over-studying, where workload and certain patterns of compulsive dedication increase exhaustion (Sanseverino et al., 2023).

In the post-pandemic period, the reconfiguration of hybrid modalities and the redesign of the academic experience have created new demands (e.g., organizational uncertainty, gaps in social support), in which appropriate teaching formats and social support have been identified as relevant predictors of engagement (Koob et al., 2021). These antecedents suggest that future studies in Chile could formally explore moderations by gender, academic trajectory, and personal resources (e.g., self-compassion, self-efficacy) to determine in which subgroups the SD-R model might show greater sensitivity (Bakker and Mostert, 2024).

## 5 Limitations

This study has several limitations that should be acknowledged. First, its cross-sectional and non-experimental design does not permit causal inferences, even though the hypothesized directions are theoretically grounded. The findings therefore indicate patterns of association consistent with SD-R theory, but they do not demonstrate temporal precedence or causation. Longitudinal and experimental studies are needed to test whether academic demands and resources prospectively influence emotional exhaustion and engagement.

Second, the use of purposive non-probability sampling limits the generalizability of the results. Although the sample size was adequate for the analyses performed, participants were drawn from a single university in northern Chile, and therefore the results should not be assumed to represent all Chilean university students or other Latin American higher education contexts. Future research would benefit from multi-institutional studies and probability-based or stratified sampling strategies that allow stronger population-level inference.

Third, all variables were measured through self-report instruments administered at a single time point, which raises the possibility of common method bias and shared source variance. Although procedural precautions were taken and the multidimensional measurement results do not support a dominant one-factor explanation, method effects cannot be ruled out completely. In addition, the study was not based on an *a priori* simulation-based power analysis, so conclusions about sample sufficiency should be interpreted in relation to model complexity and estimator performance rather than as evidence of formally established statistical power.

## 6 Conclusion

Taken together, the findings support the applicability of the SD-R/JD-R framework in Chilean higher education. In this sample, academic demands were closely associated with emotional exhaustion, whereas academic resources were positively associated with engagement, showing a pattern consistent with the health-impairment and motivational processes proposed by the theory (Demerouti et al., 2001; Lesener et al., 2020; Bakker and Mostert, 2024). Because the study was cross-sectional, these conclusions should be understood as theoretically coherent associations rather than causal effects.

The results also suggest that student well-being should not be addressed exclusively through individual coping strategies. Institutional and pedagogical conditions appear to matter substantially. In practical terms, universities may benefit from reducing avoidable hindrance demands, improving clarity in assessment and feedback processes, strengthening lecturer support, and increasing students’ sense of control and developmental opportunity. Such actions are likely to be especially valuable in contexts where students remain committed to their studies but experience moderate levels of emotional fatigue.

Overall, the study contributes to the still-limited evidence on SD-R processes in Latin American university populations and highlights the importance of examining the academic environment as a relevant correlate of student well-being. Future research should test these relationships longitudinally, compare institutions and disciplinary areas, and incorporate personal resources and contextual moderators to better understand for whom and under what conditions academic demands and resources are most strongly linked to exhaustion and engagement.

## Data Availability

The raw data supporting the conclusions of this article will be made available by the authors, without undue reservation.
